# Mapping the global threat of *Mythimna separata* (Walker) and its economic implications for Brazil's agriculture

**DOI:** 10.1002/ps.70954

**Published:** 2026-05-22

**Authors:** Gabriel Dorotel da Silva Ferreira, Cesar Augusto Marchioro

**Affiliations:** ^1^ Federal University of Santa Catarina, Campus of Curitibanos Curitibanos Brazil; ^2^ Postgraduate Program in Natural and Agricultural Ecosystems Federal University of Santa Catarina, Campus Curitibanos Curitibanos Brazil

**Keywords:** biological invasion, ecological niche modeling, invasion risk, MaxEnt, oriental armyworm, pest risk analysis

## Abstract

**BACKGROUND:**

Identifying high‐risk areas for the introduction of invasive alien species is particularly important for agricultural pests with a broad host range that attack widely cultivated crops, such as the oriental armyworm, *Mythimna separata* (Walker, 1865) (Lepidoptera: Noctuidae). This study aimed to assess the global climatic suitability for *M. separata* and evaluate the economic risk for maize, rice, sorghum, sugarcane, and wheat cultivation in Brazil in the event of invasion and spread to suitable areas. Climatic suitability was estimated using the maximum entropy (MaxEnt) algorithm, and the Normalized Concentration Index (nCI) was calculated to assess the concentration of crop production across Brazilian municipalities.

**RESULTS:**

The selected model showed strong discriminatory power, with Area Under the Curve and Continuous Boyce Index values above 0.85. Moderately to highly suitable areas were identified beyond the species' native range, including North, Central and South America, Europe, southeastern Australia, and New Zealand. Globally, 35.0%, 39.0%, and 46.9% of areas cultivated with rice, maize, and wheat, respectively, fall within regions classified as moderately to highly suitable. In Brazil, estimated economic risk varied substantially among crops, with wheat showing the greatest vulnerability.

**CONCLUSION:**

Integrating regional crop production data with invasion risk maps proved useful in identifying priority areas for preventive actions, supporting the targeting of surveillance and management efforts to mitigate potential economic losses. © 2026 The Author(s). *Pest Management Science* published by John Wiley & Sons Ltd on behalf of Society of Chemical Industry.

## Graphical abstract

Global models identified high‐risk areas for *Mythimna separata* invasion, highlighting major economic vulnerability for Brazilian crops and guiding targeted prevention and management strategies.

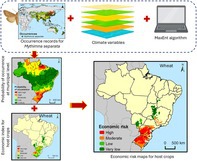

## 1 INTRODUCTION

Biological invasion refers to the introduction of a species into a new environment where it can establish, reproduce, and spread to surrounding areas.^1^ Invasive alien species are recognized as one of the greatest environmental threats, exerting profound impacts on biodiversity, provision of ecosystem services, as well as human health and the economy.^2^ They are among the main drivers of biodiversity loss and species extinction, contributing to approximately 60% of recorded global extinctions, with associated economic costs exceeding US$423 billion annually as of 2019.^2^

In Brazil, the impacts of invasive alien species are particularly severe in the agricultural sector, where accidentally introduced pests have caused substantial economic losses.^3^ Many of these exotic species, once introduced, have become important vectors of emerging diseases^4^ or agricultural pests in the invaded areas.^5^, ^6^ The economic burden of invasive pests is considerable. Globally, annual costs associated with invasive insects are estimated at US$70 billion, a significant share of which is linked to species directly affecting agricultural production.^7^ Factors such as the high connectivity of global trade and the abundance of food resources in agricultural landscapes further facilitate the introduction and spread of these pests into new regions.^5^, ^6^

The difficulties and high costs associated with eradicating and managing invasive pests make preventive measures a more cost‐effective strategy, as they enable resources to be focused on regions at greatest risk of invasion.^8^, ^9^, ^10^ Identifying such high‐risk areas requires detailed analyses of both environmental suitability and potential introduction pathways. Environmental suitability refers to the set conditions that allow a species to survive, reproduce, and establish viable populations in a given environment.^11^ Introduction pathways, in turn, are associated with factors such as logistical accessibility, the presence of ports and airports, and the intensity of international trade in a region.^6^, ^12^

Correlative ecological niche models (ENMs) have been applied to estimate areas potentially suitable for invasive species in several studies.^13^, ^14^, ^15^, ^16^ These models use occurrence records in combination with environmental variables such as climate, topography, and vegetation to predict the potential distribution of a species,^17^ including regions beyond its native range.^14^ Projections can be further refined by incorporating information on host plant availability or the economic importance of major crops, thereby enabling anticipation of the potential economic impacts of a future invasion.^16^ The Normalized Concentration Index (nCI), originally proposed to identify regional production clusters,^18^ was applied here to assess the local economic relevance of crop production.^19^ When integrated with environmental suitability data, it enables the generation of economic risk maps.^16^, ^20^

The oriental armyworm, *Mythimna separata* (Walker), is listed as a quarantine pest absent from Brazil.^21^ Native to Asia, this species is recognized for causing severe damage to major agricultural crops, including maize, rice, sorghum, sugarcane, and wheat.^22^ Its use of multiple widely cultivated crops as hosts, combined with its capacity for long‐distance migration and adaptation to diverse environmental conditions,^23^ increases the chances of dispersal into regions with environmental characteristics similar to its native range.^24^

The history of biological invasions by agricultural pests underscores the importance of studies targeting species such as *M. separata*. In this context, the present study aims to evaluate the global environmental suitability for *M. separata* and integrate this information with host crop production data for each Brazilian municipality to generate economic risk maps. We hypothesize that climatically suitable areas will be identified in multiple regions, including those where maize, rice, sorghum, sugarcane, and wheat are cultivated, highlighting the need for preventive measures to restrict the species' spread beyond its current range.

## 2 MATERIALS AND METHODS

### 2.1 Occurrence records

**Figure 1:**
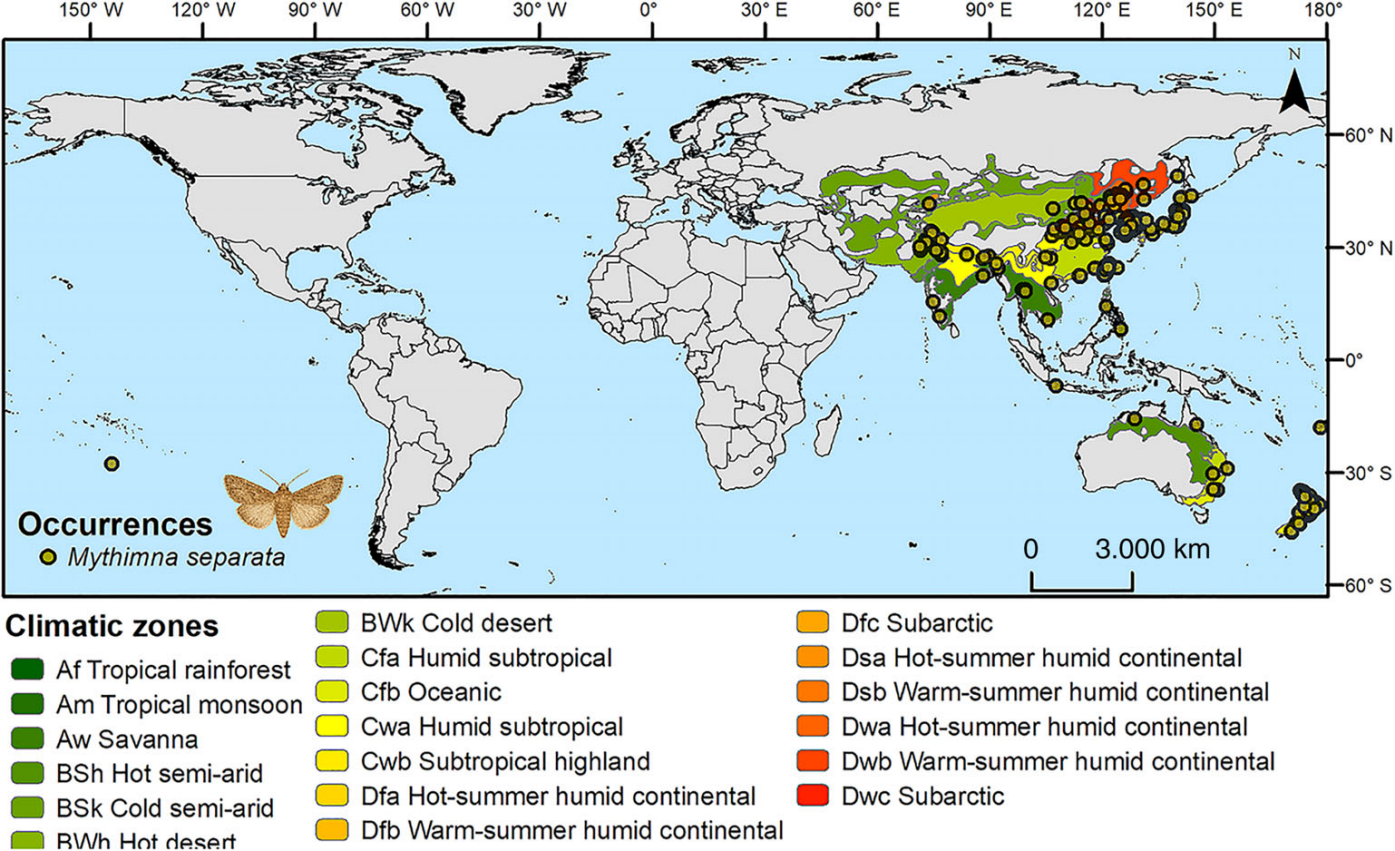
Occurrence records of *Mythimna separata* obtained from the literature and the Global Biodiversity Information Facility (GBIF) database, overlaid with the Köppen–Geiger climate zones used as background in the modeling process.

Georeferenced records of *M. separata* were obtained from the Global Biodiversity Information Facility^25^ and scientific literature; a total of 1075 records were initially compiled. To ensure data reliability, these records were cross‐checked against the species' known distribution as reported in the European and Mediterranean Plant Protection Organization database^26^ by comparing the countries where *M. separata* is reported as present and established. Occurrences with invalid coordinates, spatial duplicates, or located within 5 km of capitals or geographic centroids were removed using the R package *CoordinateCleaner*.^27^ This distance was used as a conservative buffer to remove potentially imprecise or automatically georeferenced records while minimizing the exclusion of valid occurrences. To reduce spatial autocorrelation, a minimum distance filter of 20 km between occurrence points was applied^28^ with the *spThin* package. After the data cleaning and filtering procedures, 309 unique and spatially independent occurrence records remained. The final dataset, presented in Fig. 1, reflects the species' known distribution range.

### 2.2 Background area

In presence‐only modeling algorithms, the background area represents the region where the species could potentially occur, making its delimitation a critical step in the modelling process.^29^, ^30^ In this study, the background area was delineated based on the Köppen–Geiger climate classification.^31^ Specifically, all climate zones containing at least one occurrence record of the species were selected as background area (Fig. 1). Ten thousand points randomly generated across the background area were used to characterize the environmental conditions available to the species.

### 2.3 Environmental predictors

Elevation and 14 bioclimate variables were obtained from the WorldClim database (http://worldclim.org) at a 2.5 arcminutes resolution (Table 1).^32^ These variables were derived from monthly temperature and precipitation data.^32^ Environmental predictors were clipped to the background area and tested for multicollinearity using the Variance Inflation Factor (VIF) implemented in the *usdm* package, adopting a threshold value of 10. The variables annual mean temperature (Bio1) and annual precipitation (Bio12) were retained even when collinearity was detected due to their strong ecological relevance and well‐documented influence on insect distribution patterns.

**Table 1:** Climatic variables derived from the WorldClim database used in the development of models

Variable	Code description
Bioclimatic	Bio1 – Annual mean temperature (°C)
Bioclimatic	Bio2 – Mean diurnal range (°C)
Bioclimatic	Bio4 – Temperature seasonality (standard deviation × 100)
Bioclimatic	Bio5 – Maximum temperature of warmest month (°C)
Bioclimatic	Bio6 – Minimum temperature of coldest month (°C)
Bioclimatic	Bio7 – Temperature annual range (°C)
Bioclimatic	Bio8 – Mean temperature of wettest quarter (°C)
Bioclimatic	Bio9 – Mean temperature of driest quarter (°C)
Bioclimatic	Bio12 – Annual precipitation (mm)
Bioclimatic	Bio13 – Precipitation of wettest month (mm)
Bioclimatic	Bio14 – Precipitation of driest month (mm)
Bioclimatic	Bio15 – Precipitation seasonality (coefficient of variation)
Bioclimatic	Bio18 – Precipitation of warmest quarter (mm)
Bioclimatic	Bio19 – Precipitation of coldest quarter (mm)
Topographic	Elevation (m)

### 2.4 Ecological niche modeling

The maximum entropy algorithm (MaxEnt)^33^ was applied to predict the climatic suitability for *M. separata*. MaxEnt is widely used for estimate species' potential distributions because of its strong statistical performance compared to other algorithms.^17^, ^34^ However, to ensure that models effectively discriminate between suitable and unsuitable areas, the literature recommends parameter tuning with different configurations of feature classes (i.e., transformation of covariates) and regularization multiplier.^35^, ^36^, ^37^ In this study, the *ENMeval 2.0* package^38^ was used to build 50 models combining linear, quadratic, linear‐quadratic, linear‐quadratic‐hinge, and linear‐quadratic‐hinge‐product feature classes, along with 10 regularization values ranging from 0.5 to 5.0 (in increments of 0.5). Occurrence records were partitioned using the masked structured geographic partitioning method,^36^ which divides into four spatially balanced groups (‘spatial blocks’ in *ENMeval 2.0*).

Model selection was based on three metrics: the Area Under the Curve (AUC), the Continuous Boyce Index (CBI), and the corrected Akaike Information Criterion (AICc). For AUC and CBI, values closer to 1 indicate better model performance.^17^, ^39^ In contrast, the AICc accounts for model fit and complexity, with lower values indicating superior performance. The selection process followed two steps. First, only models with test AUC and CBI values above 0.85 were retained. Among these, the final model was chosen as the one with the lowest AICc value.

To test the significance of the AUC and CBI values, we applied the null model approach proposed by Raes and Ter Steege.^40^ A total of 100 simulations were generated using random occurrences within the study area, each with the same number of records as in the empirical model and using identical model configurations. The AUC and CBI values from the empirical model were then compared with the frequency distribution obtained from the null models. The model was considered significantly better than expected by chance when the observed AUC and CBI values exceeded the 95th percentile of the null distribution.

### 2.5 Spatial analysis

#### 2.5.1 Invasion risk

The introduction probability map developed by Early *et al*.^12^ was used to identify high‐risk areas for the introduction of *M. separata*. This map was constructed based on ports and airport density, passenger flow, and international trade routes. The original map was standardized to a continuous scale ranging from 0 (low probability) and 1 (high probability). Invasion risk was then estimated by multiplying the climate suitability map generated by MaxEnt with the introduction probability map from Early *et al*.^12^

#### 2.5.2 Economic risk assessment

The 2022 production values of sugarcane, maize, rice, sorghum, and wheat for each Brazilian municipality were obtained from the Brazilian Institute of Geography and Statistics (IBGE) using the *datazoom.amazonia* package.^41^ To assess the economic relevance of each crop at the municipal level, the Normalized Concentration Index (nCI) was applied following the approach proposed by Crocco *et al*.^18^ and further detailed by Amaro *et al*.^19^, ^20^ The nCI integrates three components: (i) the specificity of crop production within a municipality, (ii) the relative importance of the crop in local agricultural production, and (iii) the municipality's contribution to national production of that crop. Thus, the nCI reflects the economic importance of each crop at the municipal scale rather than serving as a direct measure of damage or yield loss.

The location quotient (LQ) was employed to identify patterns of spatial concentration or dispersion, reflecting the degree of specialization of a municipality in the production of a given crop. High LQ values indicate greater specialization.^18^ The LQ was calculated using the following formula:

$${\mathrm{LQ}}_{\mathrm{ij}}=\left(VP_{\mathrm{ij}}/VP_{j}\right)/\left(VP_{i\mathrm{BR}}/VP_{\mathrm{BR}}\right)$$

where LQ_*ij*_ is the location quotient of crop *i* in municipality *j*, VP*ij* is the production value of crop *i* in municipality *j*, VP_*j*_ is the total agricultural production value of municipality *j*, VP_*i*BR_ is the total production value of crop *i* in Brazil, and VP_BR_ is the total agricultural production value of Brazil.

The modified Hirschman–Herfindahl index (HHI)^18^ measures the contribution of crop *i* to municipal agricultural production of municipality *j*. Positive values indicate a higher concentration of crop *i* in municipality *j*:

$$\mathrm{HH}I_{\mathrm{ij}}=\left(VP_{\mathrm{ij}}/VP_{i\mathrm{BR}}\right)-\left(VP_{j}/VP_{\mathrm{BR}}\right)$$

The third index is relative participation (RP), which evaluates the contribution of a municipality's crop production relative to the Brazilian total for that crop:

$${\mathrm{RP}}_{\mathrm{ij}}=VP_{\mathrm{ij}}/VP_{i\mathrm{BR}}$$

The nCI was calculated as the weighted sum of these three indices, with weights (*θ*) determined through principal component analysis (PCA), following the methodology described by Crocco *et al*.^18^ and Amaro *et al*.^19^, ^20^:

$${\mathrm{nCl}}_{\mathrm{ij}}=\theta_{1}LQ_{\mathrm{ij}}+\theta_{2}RP_{\mathrm{ij}}+\theta_{3}\mathrm{HH}I_{\mathrm{ij}}$$

To estimate the economic risk of invasion by *M. separata*, the probability of occurrence for each municipality was calculated based on the average climatic suitability within its boundaries. A two‐dimensional risk matrix was then applied, combining five classes of probability of occurrence (1, unlikely invasion; 2, low probability; 3, medium probability; 4, high probability; 5, very high probability) with five nCI classes (1, very low importance; 2, low importance; 3, moderate importance; 4, high importance; 5, very high importance), defined using Jenks' natural classification method. The economic risk was categorized into four levels: very low (0–1.5), low (1.5–2.0), moderate (2.0–3.0), and high (3.0–5.0). All analysis was conducted in the R environment.

#### 2.5.3 Production area at risk

To quantify the exposure of agricultural areas to *M. separata*, global‐scale geospatial data for maize, rice, and wheat were obtained at the resolution of 2.5 arcminutes.^42^ In addition, maps of sugarcane‐ and rice‐producing areas in Brazil were obtained from the MapBiomas platform at a spatial resolution of 60 m.^43^ For these crops, the percentage of agricultural crops falling within zones classified as marginally, moderately, or highly suitable for *M. separata* was calculated using spatial intersection functions in the R environment.

## 3 RESULTS

### 3.1 Model performance

**Figure 2:**
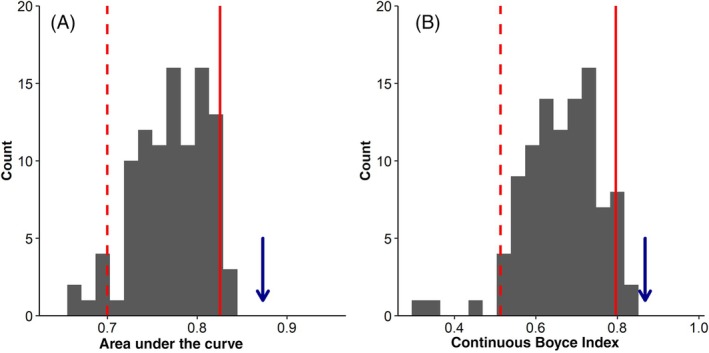
Comparison between observed (blue arrows) and simulated Area Under the Curve (A) and Continuous Boyce Index (B) values (histogram) following the null model procedure proposed by Raes and ter Steege.^40^ Dashed and solid red lines indicate the 5th and 95th percentiles of the simulated values, respectively.

The optimal model configuration for *M. separata* combined linear and quadratic feature classes with a regularization multiplier of 2 (Supporting Information, Table S1). The model showed strong discriminatory performance, with AUC values above 0.85 (AUC_TRAIN_ = 0.887, AUC_TEST_ = 0.873). Similarly, both training and test CBI (CBI_TRAIN_ = 0.917, CBI_TEST_ = 0.868) exceeded 0.85, indicating good predictive performance. Empirical AUC and CBI values were significantly higher than those obtained with the null model approach (Fig. 2).

### 3.2 Variable importance

**Figure 3:**
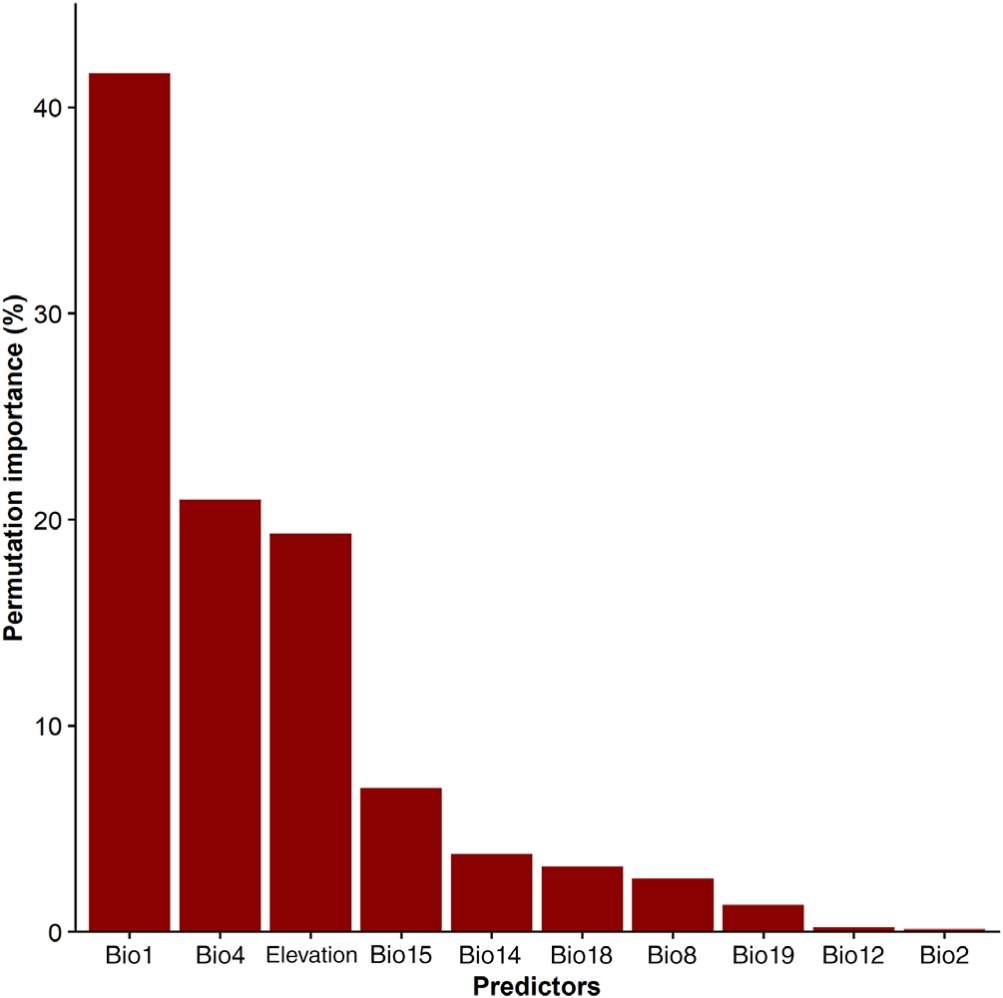
Permutation importance of the climatic variables used in the ecological niche model developed for *Mythimna separata*. Variables are annual mean temperature (Bio1), mean diurnal range (Bio2), temperature seasonality (Bio4), mean temperature of driest quarter (Bio8), annual precipitation (Bio12), precipitation of driest month (Bio14), precipitation seasonality (Bio15), precipitation of warmest quarter (Bio18), and precipitation of coldest quarter (Bio19).

**Figure 4:**
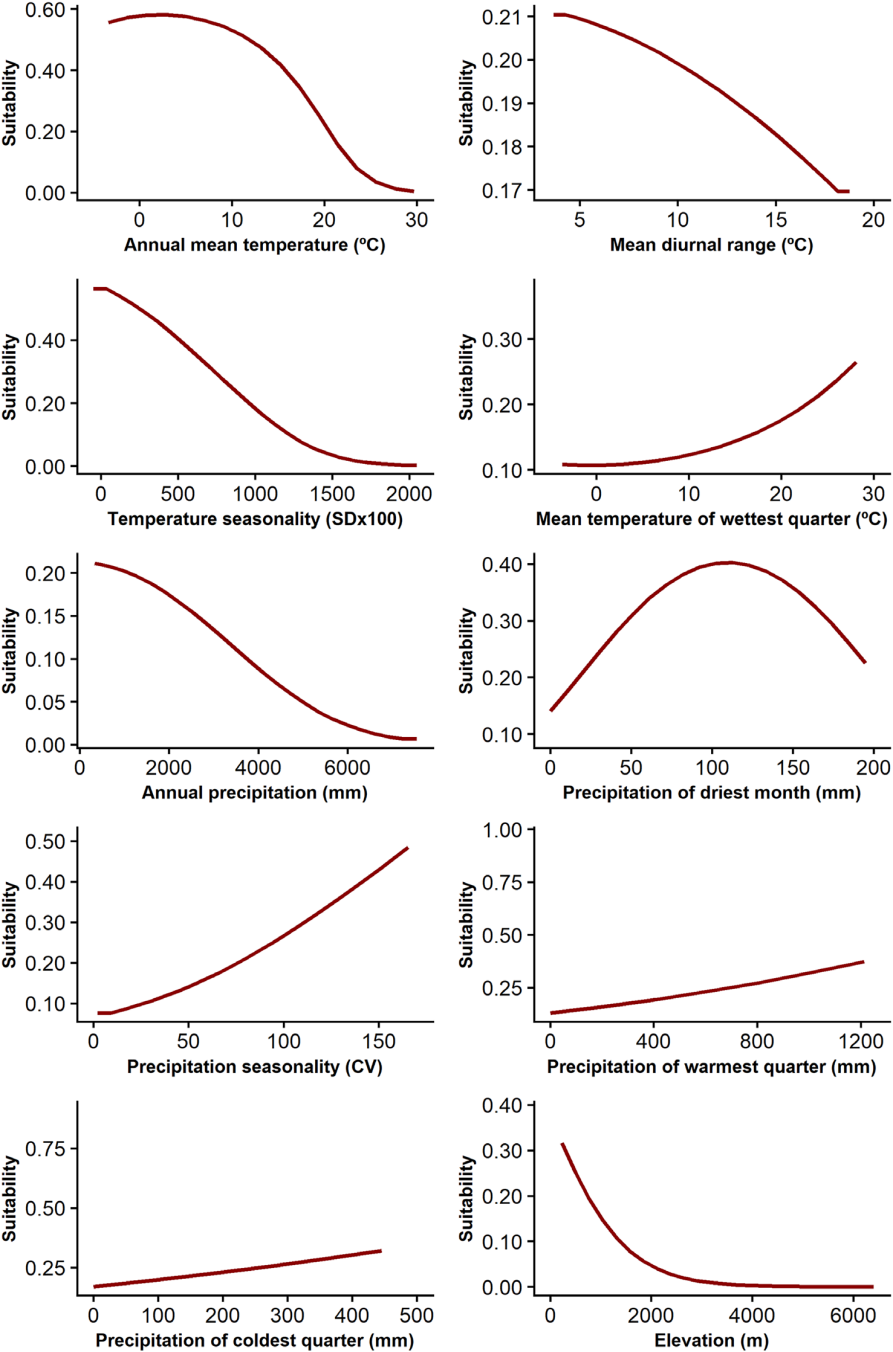
Response curves of the environmental variables used in the ecological niche model developed for *Mythimna separata*. In the plots, CV represents the coefficient of variation and SD represents the standard deviation.

Ten predictors were retained in the model after the removal of covariates: Bio1, Bio2, Bio4, Bio8, Bio12, Bio14, Bio15, Bio18, Bio19, and elevation. Among these, the variables with the greatest influence on the distribution of *M. separata* were annual mean temperature (Bio1; 41.64%), temperature seasonality (Bio4; 20.96%), elevation (19.31%), and precipitation seasonality (Bio15; 6.97%), which together accounted for 88.88% of the permutation importance (Fig. 3). Response curves indicated that species suitability increases with temperature up to approximately 15 °C but declines sharply beyond this threshold. Likewise, temperature seasonality values above 500 progressively reduced suitability. Optimal conditions were associated with low elevations (< 500 m) and high precipitation seasonality (CV > 100) (Fig. 4). Rainfall variables, such as annual precipitation (Bio12, 0.21%), contributed only marginally, confirming the species' stronger dependence on thermal and topographic variables.

### 3.3 Suitability and risk of global invasion

**Figure 5:**
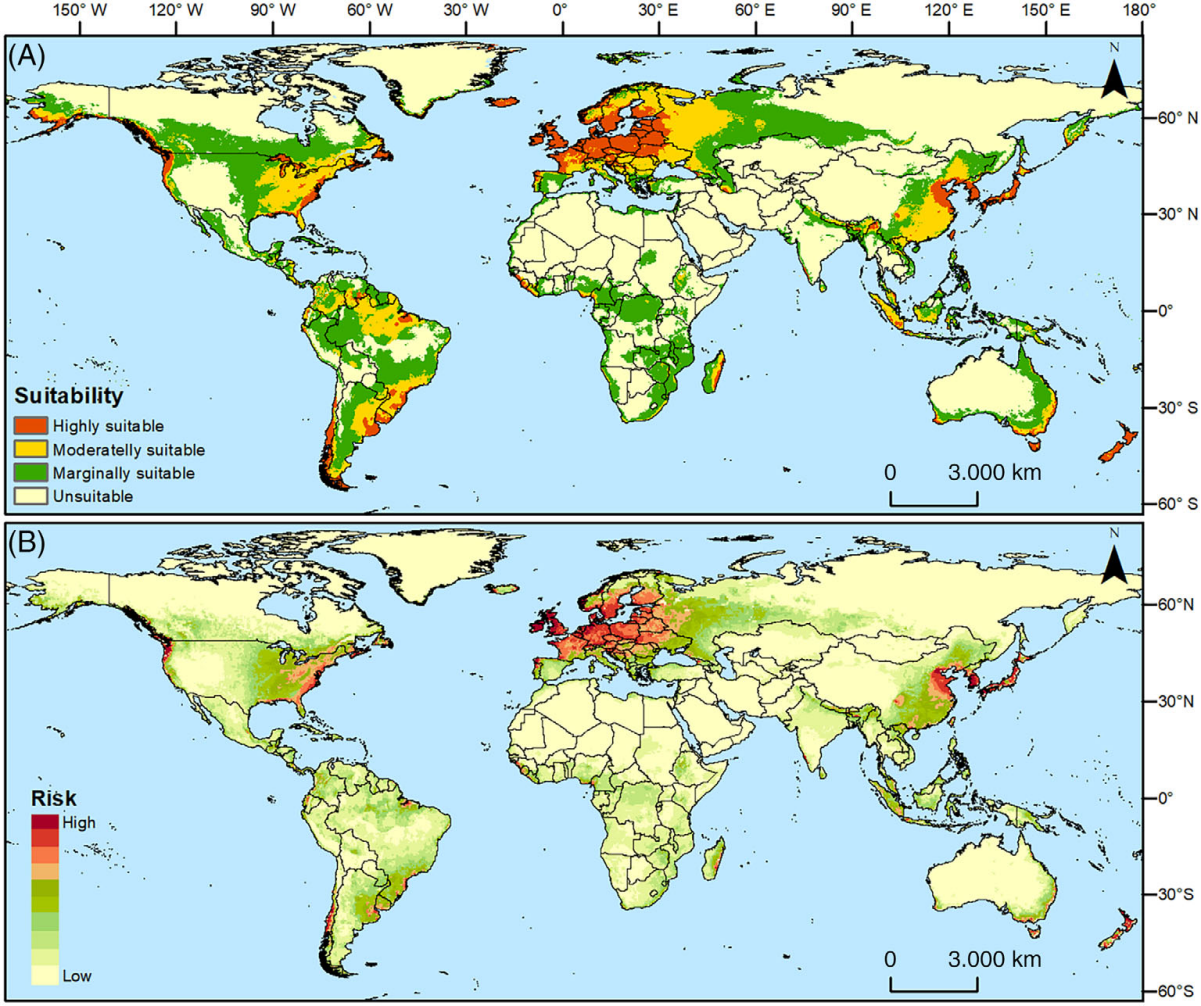
Climatic suitability maps for *Mythimna separata* generated using the MaxEnt algorithm (A) and invasion risk map (B) produced by integrating climatic suitability with probability of introduction (Early *et al*.^12^).

Moderately to highly suitable areas for *M. separata* outside its native range were identified in North America (eastern and western United States, west coast and southeast Canada), Central and South America (southern Brazil, Argentina, and Chile), various regions of Europe (extensive suitability across western and central Europe), in East Asia (China, Japan, and Korea), most of Southeast Asia, in southeastern Australia (including Tasmania), and New Zealand (Fig. 5(A)). When climate suitability was combined with the probability of introduction, areas at high risk of invasion were identified on the east coast of the United States, the west coast of North America, southeastern Canada, southern and southeastern Brazil, across western and central Europe and extending into parts of northern Europe, as well as in China and Japan, coastal areas of Southeast Asia, southeastern Australia, and New Zealand (Fig. 5(B)). Given that large portions of these regions are moderately to highly suitable, the risk of invasion by *M. separata* is considerable across multiple areas in the Americas, Europe, Asia, and Oceania.

### 3.4 Economic importance

**Figure 6:**
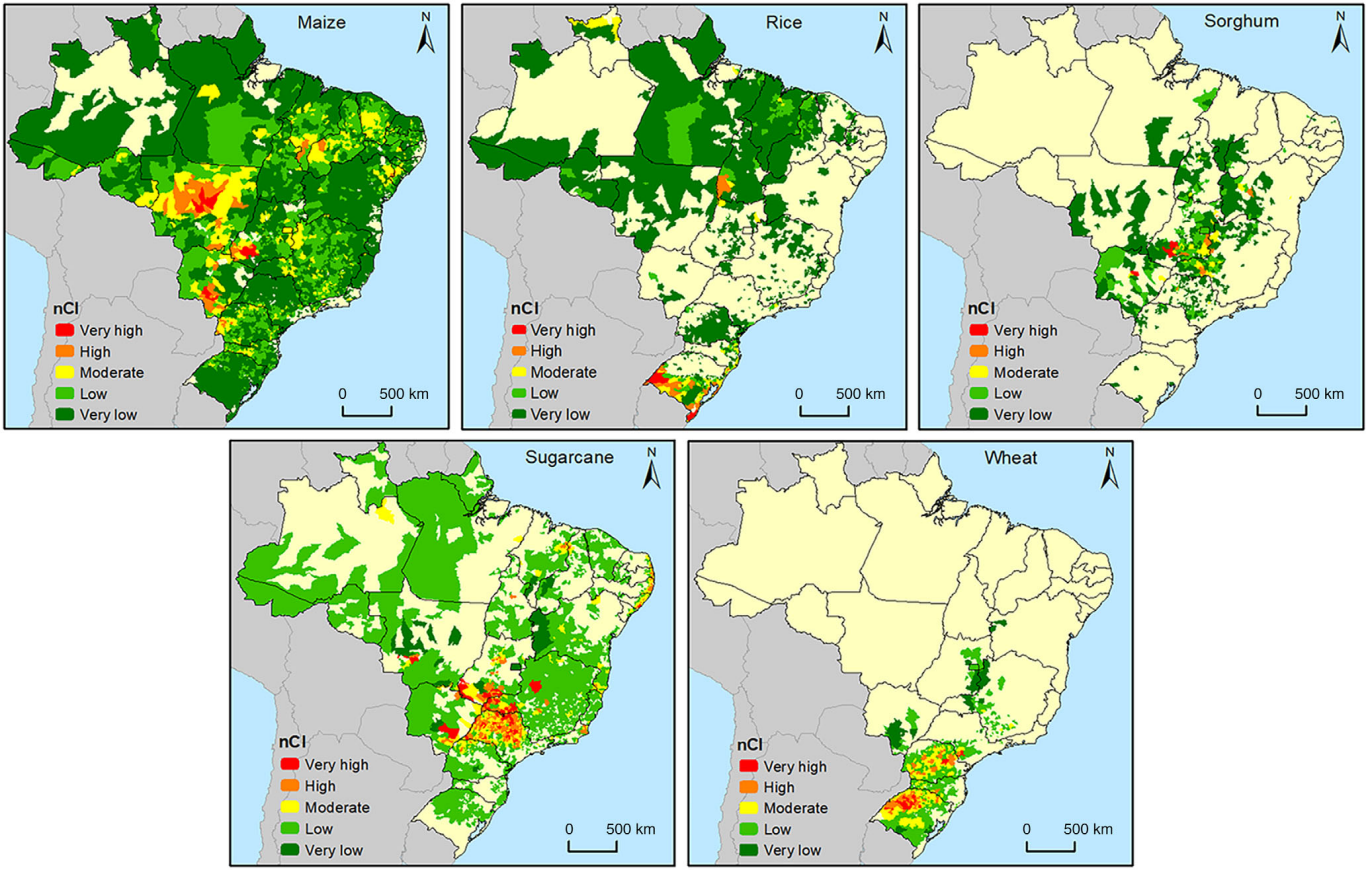
Normalized Concentration Index (nCI) for maize, rice, sorghum, sugarcane, and wheat across Brazilian municipalities. The nCI classes were derived using the natural breaks (Jenks) method, with intervals labeled to reflect the continuous distribution of the data.

The nCI calculated for each Brazilian producing municipalities of sugarcane, corn, rice, sorghum, and wheat is shown in Fig. 6. Among the 3169 municipalities producing sugarcane, 22.9% rely on this activity to a moderate to very high degree. For maize, 10.0% of the 5110 producing municipalities are moderately to highly dependent. In the case of rice, 7.1% of the 1665 producing municipalities show moderate to very high dependence. Sorghum is produced in 644 municipalities, of which 6.5% have moderate to very high dependence. Finally, the economic analysis of wheat indicates that 36.6% of the 1016 municipalities have moderate to very high dependence (Fig. 6 and Supporting Information, Table S2).

### 3.5 Municipalities at economic risk

**Figure 7:**
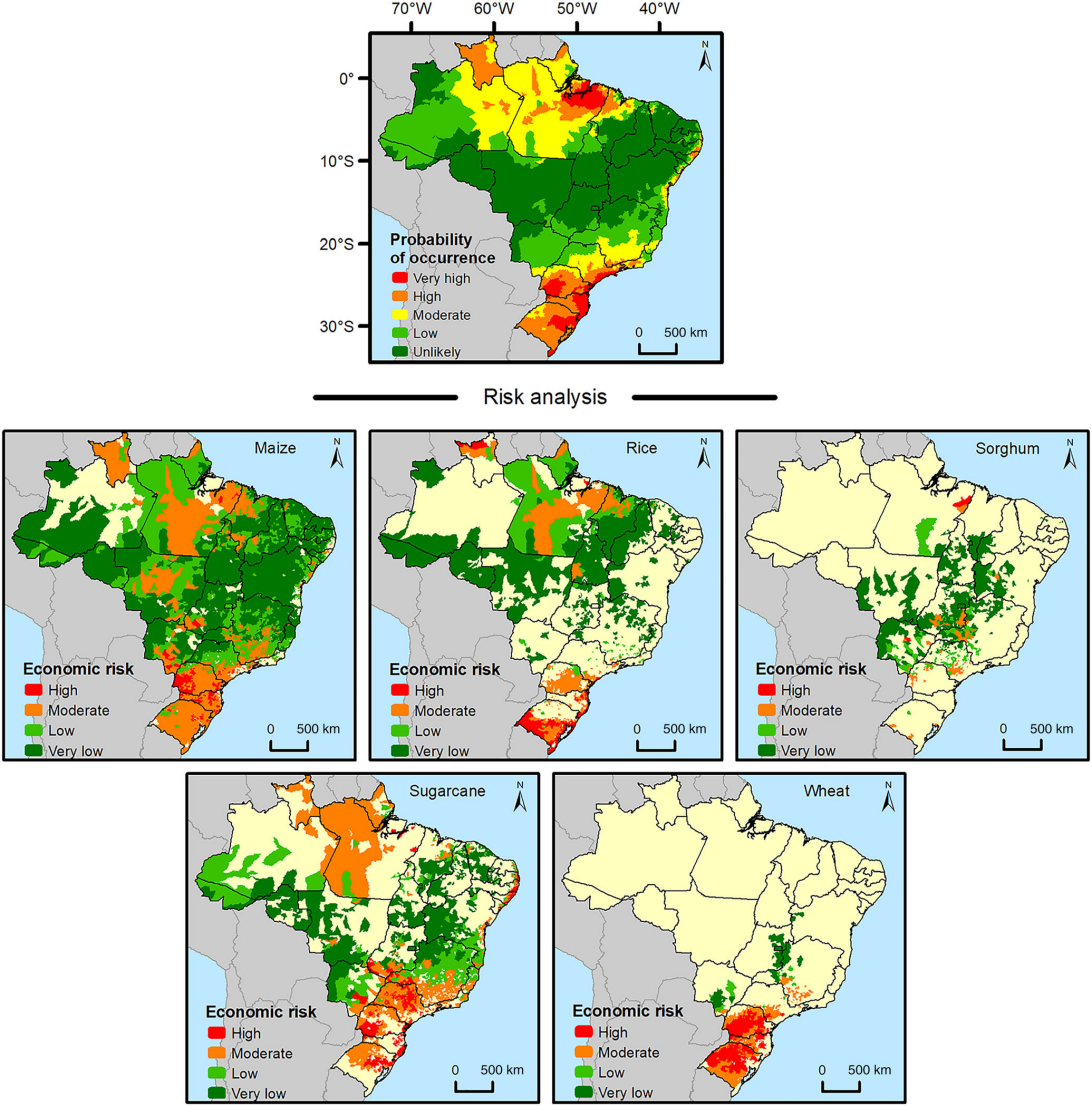
Economic risk maps for maize, rice, sorghum, sugarcane, and wheat in the event of invasion by *Mythimna separata* across Brazilian municipalities.

Figure 7 illustrates the economic risk for each evaluated crops across producing Brazilian municipalities. The number of municipalities, their location, and the degree of economic risk varied by crop (Supporting Information, Table S3). For sugarcane, 47.1% of municipalities were classified as moderate risk and 17.0% as high risk. In maize, the estimated economic risk of a biological invasion was moderate in 29.2% of the municipalities and high in 5.5%. For rice, 28.7% of municipalities faced moderate risk and 8.5% high risk. In sorghum, 15.2% of municipalities showed moderate risk and 0.3% high risk, whereas in wheat, the risk was 45.8 and 48.9%, respectively (Supporting Information, Table S3). Across all crops, municipalities at moderate to high risk were concentrated mainly in the south and southeast regions, reflecting the overlap between climatic suitability and the distribution of producing areas.

The overlap between crop production areas and climatically suitable zones for *M. separata* revealed distinct patterns among crops. For rice 38.8% of cultivated areas were located in marginally suitable zones, 27.4% in moderately suitable zones, and 7.6% in highly suitable zones. For maize, 40.6% of the production areas overlapped with marginally suitable zones, 27.3% with moderately suitable zones, and 11.7% with highly suitable zones. Wheat‐growing areas showed 38.6% overlap with marginally suitable zones, 28.7% with moderately suitable zones, and 18.2% with highly suitable zones. In Brazil, 90.7% of rice and 36.2% of sugarcane production areas were found within climatically suitable zones for *M. separata* (Fig. 7).

## 4 DISCUSSION

Identifying areas at risk of invasion by exotic species is essential for designing effective preventive strategies.^9^, ^11^ In this context, climate suitability maps generated by ENMs provide an initial indication of where a species may establish if introduced. Preventive planning can be further refined by incorporating regional information on potential entry routes and the availability of host plants.^14^, ^16^ In this study, climate suitability data for *M. separata* were integrated with the spatial distribution and economic importance of maize, rice, sorghum, sugarcane, and wheat in Brazil. The results revealed that extensive producing areas are located within suitable zones for *M. separata*. Moreover, the economic assessment highlights that this pest poses a significant threat to the economy of several Brazilian municipalities.

The reliability of projections generated by ENMs depends on the models' ability to capture the species' biology and ecology to accurately predict its distribution. The statistical metrics applied indicate that the model successfully distinguished between suitable and unsuitable habitats for *M. separata*. The response curves highlight mean annual temperature and temperature seasonality as the most critical determinants, together explaining 62.6% of the species' distribution. Suitability increases with temperature up to a peak around 15 °C, declining steadily at higher temperatures. Indeed, high temperatures are known to cause elevated pupal mortality and reduce female fecundity in *M. separata*.^44^ Elevation emerged as the third most influential factor, with suitability highest at low elevations (<500 m) and declining steeply as elevation increases, exhibiting an approximately exponential decay and approaching negligible values at high altitudes. This pattern is consistent with field and experimental studies showing that warmer lowland thermal regimes, greater availability/concentration of host plants, and favorable flight conditions at lower elevations enhance survival, reproduction, and migratory aggregation of *M. separata*.^45^, ^46^

Several regions outside the native range of *M. separata* have been identified as climatically suitable, including southern and southeastern Brazil, the eastern United States, and Western Europe. Many of these areas are close to major ports and international trade hubs, increasing the chances of introduction through commodity transport^6^ and underscoring the need of priority surveillance and management efforts. In Brazil, the historical expansion of maize, sugarcane, and wheat, currently covering 9.94 million hectares,^47^ has created critical vulnerability hotspots, with 36.2% of sugarcane fields overlapping climatically suitable zones for the pest. Extensive monocultures with high plant densities, combined with inadequate management practices, further elevate the risk of establishment.^48^, ^49^, ^50^ Agricultural practices such as limited crop rotation and high plant densities replicate ideal conditions for outbreaks, similar to those reported in Asia, where severe losses have been recorded.^51^

The extensive cultivation of maize, rice sorghum, sugarcane, and wheat, combined with the long history of biological invasions by insect pests and their associated economic losses, underscores concern about the potential spread of *M. separata* to new regions. In this context, prioritizing areas based on regional characteristics is essential for producing risk maps that can guide the development of effective preventive measures. Following the methodology proposed by Amaro *et al*.,^19^ a two‐dimensional risk matrix was applied to generate risk maps that integrate the probability of *M. separata* occurrence with the regional economic importance of host crops. A key strength of this approach is its ability to produce invasion risk maps at the municipal level, an administrative scale that facilitates the implementation of actions by phytosanitary authorities.^16^ Furthermore, recognizing the potential economic losses associated with the *M. separata* invasion allows anticipation of social challenges in municipalities heavily dependent on the attacked crops.

The high dispersal capacity of *M. separata* is a key factor facilitating its spread across suitable areas in the event of invasion. Radar studies show that this species migrates at altitudes of 50–500 m, at speeds ranging from 4 to 12 m/s, flying about 10 h per night.^23^ This enables displacements of up to 144 km per night, meaning that once introduced, *M. separata* could rapidly spread to climatically suitable and economically vulnerable regions. This mobility is confirmed by genetic analyses of Chinese populations, which reveal high gene flow, low genetic differentiation, and no correlation between geographic distance and genetic distance, evidence of frequent migrations and interconnectivity between distant populations.^52^ This ability highlights the importance of surveillance at possible points of entry. In contrast, there is currently no evidence of accidental introductions *of M. separata* via the transport of plant material containing larvae, pupae, or adults, suggesting that natural aerial migration remains its primary dispersal pathway. Nevertheless, accidental introduction could still occur, albeit less likely, through agricultural residues and by‐products associated with trade and transport (e.g., straw, stalks, grain bags) that may host immature stages. For this reason, mapping climatically suitable areas alongside regions of high economic dependence remains essential, as the potential damage caused by *M. separata* invasion would far outweigh the costs of prevention.

The regional economic risk maps produced in this study have direct implications for phytosanitary surveillance and early‐detection systems. These maps support the prioritization and allocation of limited surveillance resources by guiding routine inspections and monitoring efforts towards ports of entry and municipalities identified as priority areas.^12^, ^53^ Practical monitoring measures may include targeted inspection of agricultural products and residues, the establishment of trap networks in high‐priority municipalities, and the implementation of standardized reporting procedures and contingency plans at the municipal level. Integrating municipal economic risk maps with existing phytosanitary services can facilitate earlier detection and more coordinated responses, thereby increasing the likelihood of successful containment or eradication of newly detected introductions.

Historical records of invasive pests in Brazil illustrate both the limitations and the potential of coordinated management responses. When the Old World bollworm *Helicoverpa armigera* (Hübner) was first detected in Brazil in 2013, it was already widely distributed across major agricultural production, causing substantial economic losses and making eradication unfeasible.^54^ In addition to its morphological similarity to *Helicoverpa zea* (Boddie), a pest commonly found in Brazil, genetic analysis indicates that *H. armigera* invaded the country through multiple independent introduction events.^55^ In contrast, the codling moth *Cydia pomonella* (L.) was detected at an early stage in Brazil and subjected to a coordinated eradication programme that combined detection trapping, host removal, and coordinated local management, culminating in a formal declaration of eradication for the pest in 2014.^55^, ^56^, ^57^ Early detection, knowledge of climatically suitable areas, and a rapid, well‐resourced response were decisive in preventing large‐scale spread, highlighting the critical role of surveillance and early‐warning systems. These contrasting outcomes underscore two practical lessons for *M. separata*: early detection followed by rapid, well‐resourced and coordinated interventions can enable eradication when populations remain spatially limited, whereas widely established polyphagous pests tend to require long‐term management and higher economic costs.

## 5 CONCLUSION

In this study, global invasion risk maps were developed for *M. separata* by integrating climatic suitability with the probability of introduction, revealing that several regions beyond its native range exhibit moderate to high invasion risk. The analysis was subsequently refined for the Brazilian context by incorporating data on the production value of maize, rice, sorghum, sugarcane, and wheat to quantify their regional economic relevance through the nCI. Using a two‐dimensional risk matrix that combines the probability of occurrence and nCI values for different crops, an economic risk map was generated at the municipal scale. The results indicate that a substantial proportion of producing municipalities face moderate to high economic risk, particularly in the south and southeast regions, where climatic suitability coincides with strong economic dependence on these crops. These results provide a robust framework to guide preventive management strategies against this potential pest and to anticipate economic impacts in the event of its establishment.

## FUNDING INFORMATION

This research was financially supported by the Brazilian National Council for Scientific and Technological Development (CNPq) (project number 302201/2025‐4).

## Supporting information


**Table S1.** Performance metrics of the models developed for *Mythimna separata*. The selected model is highlighted in bold.
**Table S2.** Percentage of producing municipalities of maize, rice, sorghum, sugarcane, and wheat in each nCI class.
**Table S3.** Percentage of producing municipalities of maize, rice, sorghum, sugarcane, and wheat in each economic risk class.

## Data Availability

The data that support the findings of this study are available from the corresponding author upon reasonable request.
