# Supplementary material for: Mapping the global threat of Mythimna separata (Walker) and its economic implications for Brazil's agriculture
Source: Pest Manag Sci. 2026 May 22;82(9):9097–106. doi: 10.1002/ps.70954 (PMC13453250; doi:10.1002/ps.70954)
Supplement: Supplementary file 1 [file PS-82-9097-s001.pdf]

Mapping the global threat of *Mythimna separata* (Walker) and its economic implications for Brazil's agriculture

Gabriel Dorotel da Silva Ferreira[a], Cesar Augusto Marchioro[b]*

[a] Federal University of Santa Catarina, Campus of Curitibanos, Santa Catarina, Brazil. E-mail: gabriel.dorotel@gmail.com

[b] Postgraduate Program in Natural and Agricultural Ecossystems, Federal University of Santa Catarina, Campus Curitibanos, Santa Catarina, Brazil. E-mail: cesmarc@gmail.com

* Corresponding author

Table S1. Performance metrics of the models developed for *Mythimna separata*. The selected model is highlighted in bold.

FC[a]	RM[b]	AUC_{Train}[c]	CBI_{Train}[d]	AUC_{Test}	CBI_{Test}	AICc[e]	ΔAICc[f]
L	0.5	0.863	0.936	0.863	0.819	4502.289	130.939
Q	0.5	0.872	0.969	0.873	0.833	4485.217	113.868
LQ	0.5	0.890	0.931	0.851	0.519	4371.350	0.000
LQH	0.5	0.940	0.993	0.827	0.566	5395.131	1023.781
LQHP	0.5	0.940	0.988	0.820	0.630	5392.575	1021.226
L	1	0.863	0.938	0.863	0.809	4504.685	133.336
Q	1	0.872	0.972	0.877	0.833	4486.545	115.195
LQ	1	0.889	0.913	0.863	0.669	4376.613	5.264
LQH	1	0.930	0.978	0.845	0.518	4578.136	206.786
LQHP	1	0.931	0.990	0.841	0.684	4600.247	228.897
L	1.5	0.863	0.943	0.863	0.780	4508.482	137.133
Q	1.5	0.872	0.983	0.878	0.881	4490.890	119.540
LQ	1.5	0.887	0.915	0.870	0.841	4382.237	10.887
LQH	1.5	0.923	0.991	0.855	0.547	4481.797	110.448
LQHP	1.5	0.926	0.993	0.844	0.530	4501.794	130.444
L	2	0.862	0.960	0.862	0.765	4513.801	142.452
Q	2	0.871	0.981	0.878	0.887	4493.899	122.550
**LQ**	**2**	**0.887**	**0.917**	**0.873**	**0.868**	**4385.915**	**14.565**
LQH	2	0.920	0.970	0.863	0.665	4480.933	109.583
LQHP	2	0.921	0.975	0.846	0.482	4461.917	90.568

L	2.5	0.861	0.984	0.859	0.780	4519.974	148.624
Q	2.5	0.871	0.972	0.877	0.863	4497.823	126.473
LQ	2.5	0.886	0.913	0.876	0.881	4390.838	19.488
LQH	2.5	0.916	0.948	0.864	0.640	4402.092	30.743
LQHP	2.5	0.916	0.981	0.854	0.517	4472.603	101.254
L	3	0.859	0.987	0.853	0.714	4527.062	155.712
Q	3	0.871	0.973	0.875	0.826	4502.971	131.622
LQ	3	0.886	0.921	0.878	0.888	4394.925	23.576
LQH	3	0.911	0.935	0.866	0.620	4395.189	23.840
LQHP	3	0.911	0.977	0.853	0.573	4440.134	68.785
L	3.5	0.857	0.979	0.847	0.708	4535.250	163.900
Q	3.5	0.870	0.980	0.872	0.779	4509.554	138.205
LQ	3.5	0.885	0.920	0.881	0.874	4401.995	30.646
LQH	3.5	0.906	0.943	0.867	0.636	4407.089	35.739
LQHP	3.5	0.905	0.978	0.855	0.577	4409.268	37.919
L	4	0.855	0.980	0.840	0.713	4544.098	172.748
Q	4	0.869	0.985	0.868	0.760	4514.545	143.196
LQ	4	0.885	0.933	0.882	0.863	4408.578	37.228
LQH	4	0.903	0.974	0.870	0.716	4403.416	32.066
LQHP	4	0.901	0.968	0.859	0.604	4402.338	30.989
L	4.5	0.852	0.981	0.834	0.667	4551.429	180.079
Q	4.5	0.868	0.983	0.864	0.731	4515.348	143.998
LQ	4.5	0.884	0.932	0.883	0.868	4411.387	40.038
LQH	4.5	0.900	0.974	0.875	0.805	4396.337	24.988
LQHP	4.5	0.898	0.962	0.859	0.640	4392.824	21.475
L	5	0.850	0.983	0.830	0.649	4561.243	189.893
Q	5	0.867	0.982	0.860	0.704	4519.761	148.411
LQ	5	0.884	0.940	0.884	0.878	4416.426	45.077
LQH	5	0.899	0.972	0.876	0.859	4418.737	47.387
LQHP	5	0.897	0.957	0.853	0.625	4385.118	13.768

[a] Feature classes: L – linear, Q – quadratic, H – hinge, P – product.

[b] Regularization multiplier values.

[c] Area Under the Curve of the Receiver Operating Characteristics.

[d] Continuous Boyce Index.

[e] corrected Akaike Information Criterion.

[f] Difference between the AICc obtained by the model and the lowest AICc obtained.

Table S2 Percentage of producing municipalities of maize, rice, sorghum, sugarcane, and wheat in each *nCI* class.

Economic risk class	Maize	Rice	Sorghum	Sugarcane	Wheat
Very low	42.0	49.1	60.6	18.4	3.6
Low	23.4	13.6	23.2	17.5	1.7
Moderate	29.2	28.7	15.2	47.1	45.8
High	5.5	8.5	0.3	17.0	48.9

Table S3 Percentage of producing municipalities of maize, rice, sorghum, sugarcane, and wheat in each economic risk class.

nCI classes	Maize	Rice	Sorghum	Sugarcane	Wheat
Very low	60.9	81.8	75.3	0.9	3.1
Low	29.1	11.1	18.2	76.2	60.2
Moderate	9.0	5.7	4.8	14.6	26.7
High	0.9	1.2	1.2	6.8	8.3
Very high	0.1	0.2	0.5	1.5	1.7
